# Mandibular Movements As Accurate Reporters of Respiratory Effort during Sleep: Validation against Diaphragmatic Electromyography

**DOI:** 10.3389/fneur.2017.00353

**Published:** 2017-07-21

**Authors:** Jean-Benoît Martinot, Nhat-Nam Le-Dong, Valerie Cuthbert, Stephane Denison, Philip E. Silkoff, Hervé Guénard, David Gozal, Jean-Louis Pepin, Jean-Christian Borel

**Affiliations:** ^1^CHU UCL Namur, Namur, Belgium; ^2^RespiSom, Erpent, Belgium; ^3^Penn Valley, Allentown, PA, United States; ^4^Université and CHU de Bordeaux, Bordeaux, France; ^5^University of Chicago, Chicago, IL, United States; ^6^CHU de Grenoble, Laboratoire EFCR, Pôle Thorax et Vaisseaux, Grenoble, France; ^7^University Grenoble Alps, HP2 INSERM U1042, Grenoble, France; ^8^AGIR à dom. Association, Meylan, France

**Keywords:** obstructive sleep apnea, respiratory effort, mandibular movements, polysomnography, diaphragm electromyography

## Abstract

**Context:**

Mandibular movements (MM) are considered as reliable reporters of respiratory effort (RE) during sleep and sleep disordered breathing (SDB), but MM accuracy has never been validated against the gold standard diaphragmatic electromyography (EMG-d).

**Objectives:**

To assess the degree of agreement between MM and EMG-d signals during different sleep stages and abnormal respiratory events.

**Methods:**

Twenty-five consecutive adult patients with SDB were studied by polysomnography (PSG) that also included multipair esophageal diaphragm electromyography and a magnetometer to record MM. EMG-d activity (microvolt) and MM (millimeter) amplitudes were extracted by envelope processing. Agreement between signals amplitudes was evaluated by mixed linear regression and cross-correlation function and in segments of PSG including event-free and SDB periods.

**Results:**

The average total sleep time was 370 ± 18 min and the apnea hypopnea index was 24.8 ± 5.2 events/h. MM and EMG-d amplitudes were significantly cross-correlated: median *r* (95% CI): 0.67 (0.23–0.96). A mixed linear model showed that for each 10 µV of increase in EMG-d activity, MM amplitude increased by 0.28 mm. The variations in MM amplitudes (median range: 0.11–0.84 mm) between normal breathing, respiratory effort-related arousal, obstructive, mixed, and central apnea periods closely corresponded to those observed with EMG-d activity (median range: 2.11–8.23 µV).

**Conclusion:**

MM amplitudes change proportionally to diaphragmatic EMG activity and accurately identify variations of RE during normal sleep and SDB.

## Introduction

Accurately assessing respiratory effort (RE) is a critically important factor in the context of identifying obstructive and central events in sleep breathing disorders (SDB). The American Academy of Sleep Medicine (AASM) recommends as gold standard the use of esophageal manometry that directly and quantitatively reflects variations in intrathoracic pressures (1). This measurement is considered as the reference standard for assessing RE but is under the influence of the thoracic volume, and a range of other potential masking factors such as chest wall dynamics, airway resistance, lung compliance, and respiratory muscle activity (2–4). Moreover, the placement of an esophageal catheter is often uncomfortable and unacceptable for patients, may modify upper airway dynamics, and impair sleep quality and reduce its duration (2, 5–7).

Respiratory inductive plethysmography (RIP) measures the temporal coordination of thoracic and abdominal movements. Phase differences of thoracoabdominal movements (i.e., asynchrony) could reflect RE required to overcome upper airway obstruction during sleep. However, there are still some concerns about the reliability of RIP particularly in obese patients due to the role of adiposity in the induction of artifacts and associated decreases in signal response. Moreover, excursions in the thorax or abdominal belts can vary with body position, and there is a risk of misclassification of obstructive apneas (1, 8). Ideally, less invasive, more practical, reliable, and more cost-effective techniques for investigating patients with suspected SDB are sorely needed (9).

Mandibular movements (MM) patterns are a sensitive and specific approach to identify REs related to obstructed breaths in obstructive sleep apnea (OSA) (10–14). However, the performance of MM has never been validated against the reference measurement of the diaphragmatic EMG activity recorded by electrodes inserted on an endo-esophageal catheter diaphragmatic electromyography (EMG-d). Esophageal recordings of EMG-d have been used frequently to evaluate diaphragm activation in normal individuals and in patients with respiratory diseases (15–17). The EMG-d signal amplitude is closely related to global diaphragm activation and is not influenced by changes in chest wall configuration or lung volume resistance or compliance (18). During sleep-associated apneic events, EMG-d activity truly represents the neural respiratory drive and the level of RE (19).

The primary aim of this study was to examine the relationship between the changes in MM signal amplitudes and the change in tidal EMG-d activity during normal sleeping patterns in sleeping individuals and across different profiles of SDB among patients with obstructive sleep apnea syndrome (OSAS).

## Materials and Methods

### Study Subjects

Thirty consecutive adult patients, referred for suspected OSAS in a single sleep center (University Hospital Namur, Belgium) were invited to participate. All participants had symptoms suggestive of underlying SDB. The study was approved by the local human ethics committee (IRB 00004890—No. B707201523388), and all participants provided a written informed consent.

### Study Design

This was a prospective cross sectional study performed during a single night polysomnography (PSG).

### Measurements and Data Acquisition

#### Polysomnography

In-laboratory PSG was recorded with a commercial digital acquisition system (Somnoscreen Plus, Somnomedics, Randersacken, Germany). The parameters monitored included Electroencephalogram (Fz-A+, Cz-A+, Pz-A+), right and left electroocculogram, submental EMG, tibial EMG, chest, and abdominal wall motion by respiratory inductance plethysmography (SleepSense S.L.P. Inc., St. Charles, IL, USA), nasal and oral flows, respectively, with a pressure transducer and a thermistor, and O_2_ saturation by digital oximeter displaying pulse wave form (Nonin, Nonin Medical, Plymouth, MN, USA).

#### Mandibular Movements

**Figure 1:**
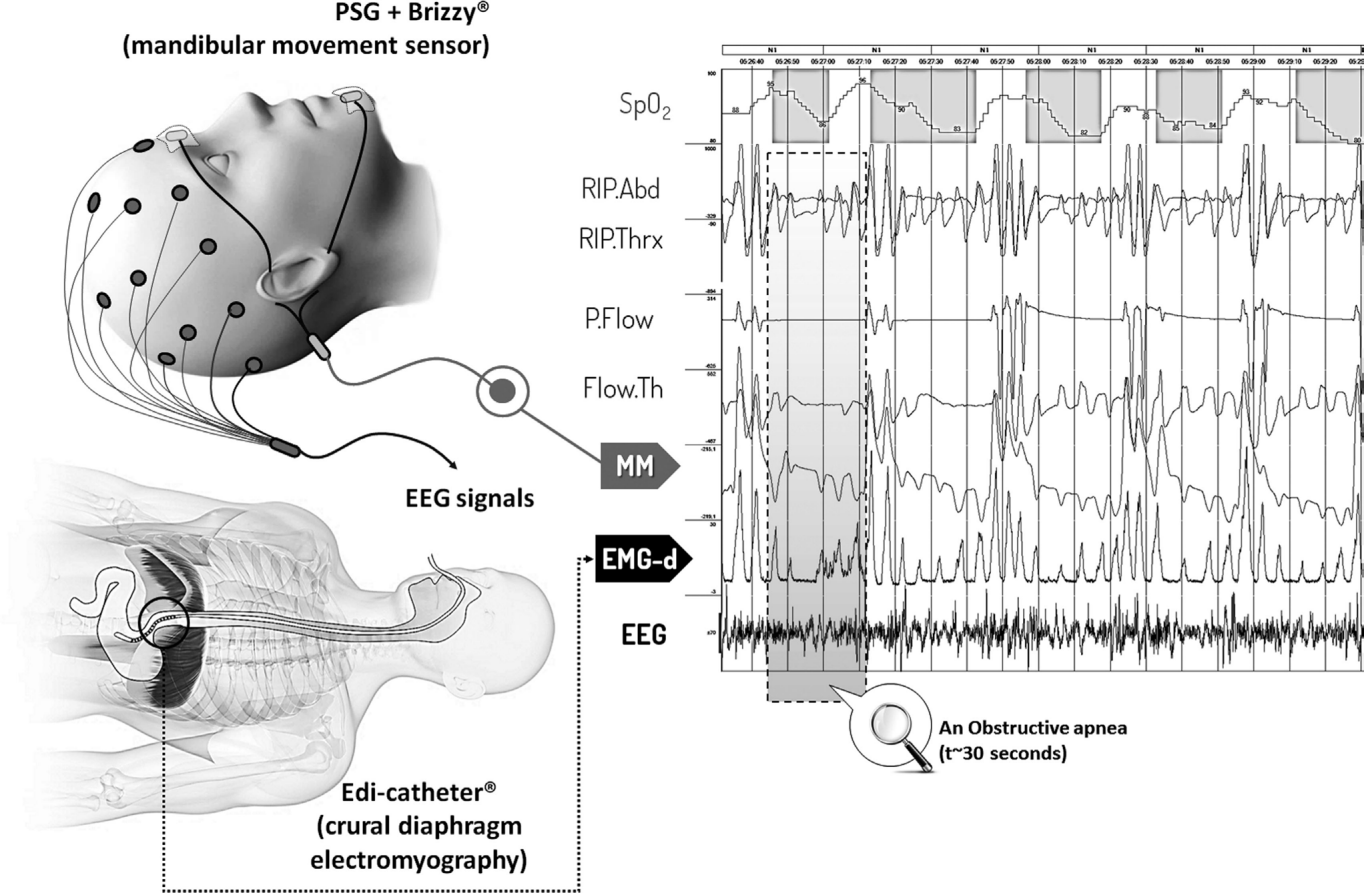
Polysomnographic 3 min segment including Mandibular movements and diaphragmatic electromyography (EMG-d) signals during episodes of obstructive apnea and hypopnea. Abbreviations: SpO_2_, oxygen saturation; RIP. Abd, RIP. Thx, abdominal and thoracic inductance belts; P. Flow, Flow. Th, nasal pressure transducer and oronasal thermal flow sensor; MM, mandibular movements; EMG-d, diaphragmatic electromyography; EEG, electro-encephalogram derived signal (C4:A1).

Mandibular movements were assessed with a midsagittal MM magnetic sensor (Brizzy^®^ Nomics, Liege, Belgium), which measures the distance between two parallel, coupled, resonant circuits placed on the forehead and on the chin. It was used to record MMs (20). The transmitter generates a pulsed magnetic wave of low energy. The change in the magnetic field recorded at the receiver is inversely related to the cube of the distance between the chin and forehead probes. The distance between two probes is measured in millimeter with a resolution of 0.05 mm. Basically, this signal provides the instantaneous position of the mandible (Figure 1).

#### Diaphragmatic Electromyography

EMG-d signals were obtained *via* a multiple array endo-esophageal electrode (Edi-catheter^®^ Getinge, SWE) consisting in nine stainless steel rings placed 10 mm apart creating an array of eight sequential differential bipolar electrodes pairs mounted on silicone tubing. The most caudal pair of rings was considered as the reference electrode. The raw EMG-d signal obtained with multiple pair electrode array is a “surface recording” from crural diaphragm motor units. The crural diaphragm forms a muscular tunnel that covers the esophagus and the muscle fibers are mostly perpendicular to the catheter. It has been demonstrated that bipolar EMG electrodes oriented perpendicularly to the muscle fiber direction are adequately positioned for recording of EMG-d (15, 16). The appropriate position of the multiple-array endo-esophageal electrode was controlled by an automatic computer algorithm that eliminates the influence of the ECG motion artifacts and background noise. The algorithms continuously calculate the RMS value for sequential EMG segments of 50 ms duration. In this way, the EMG-d signal is integrated over a period of 50 ms.

EMG signals were incorporated into the PSG montage as root-mean-square values keeping the same unit than the original signal (microvolts) (21, 22).

#### PSG Scoring

Polysomnographic scoring (sleep stages and respiratory events) was performed by two trained technicians who were blinded to the study aims and according to the criteria of Rechtschaffen and Kales and the AASM rules, respectively (1). Normal breathing periods and five types of sleep disordered breathing (SDB) events were scored: Respiratory Effort Related Arousals (RERAs), hypopneas, obstructive apneas, central apneas, and mixed apneas. Analysis was restricted to 25 of the 30 originally recruited patients who spent a minimum of 4 h sleeping along with good quality signals on all recorded channels.

#### Data Processing

##### Signal Processing

**Figure 2:**
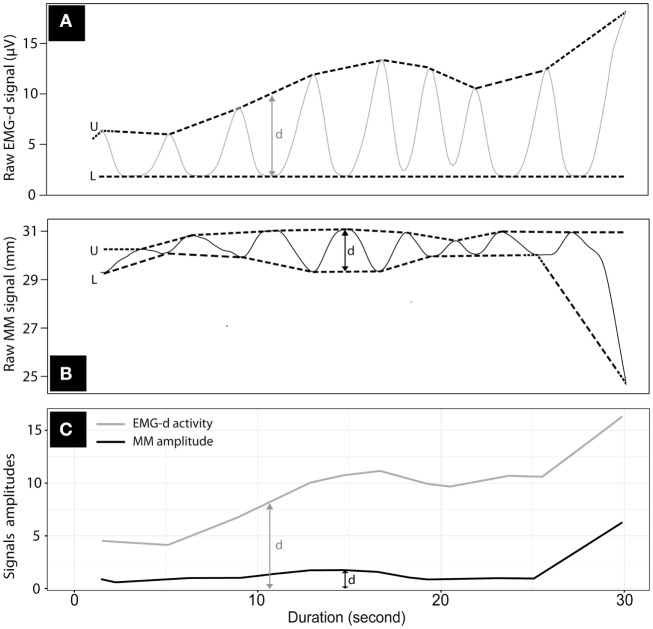
Computerized envelop processing around the traces of Mandibular movements (MM) and diaphragmatic electromyography (EMG-d) over 30 s. Envelop processing on raw signals of EMG-d **(A)** and MM **(B)**; U = Upper-band; L = Lower band; d = amplitude, determined as the difference between upper and lower bands; **(C)** traces of enveloped EMG-d (gray line) and MM (black) amplitudes.

The procedure for EMG-d and MM signals computerized processing is displayed in Figure 2: portions of signals including micro-arousals non-related to respiratory events and awakening periods were excluded from the computerized processing. After noise reduction and resampling at 10 Hz, individual datasets underwent envelope treatment around signals to capture their amplitudes and build the final database (23).

The noise reduction procedures consisted of a time series decomposition. This mathematical approach aimed to split a time series into three components: seasonality, trends and random (noise). The seasonal component detected signal patterns that repeated with fixed period of time. Trend extracted the underlying trend of the metrics (example: the MM and EMG-d amplitudes would increase during an obstructive apnea). Random (also called “noise”) consisted of the residuals of the time series after allocation into the seasonal and trends. Finally, we reconstructed a new time series in which all the random noises were excluded. The envelope treatment comprised predetermined timely signal splitting in segments from which only maximal and minimal values were computed.

##### Statistical Analysis

First, cross-correlations between MM and EMG-d signals amplitudes were performed by applying a cross-correlation function on bootstrapped segments of equal-sized data for each one of the five different types of SBD and periods of normal breathing (24, 25). The coefficients were then averaged for all events.

Then, a random slope linear regression analysis considering MM as response variable and EMG-d interacting with REM sleep as predictors was performed (26). The model recaptured fixed effects of REM and EMG-d amplitudes as well as individual random effects on both intercept and slopes.

Finally, a pairwise quantile comparison of the medians of MM and EMG-d amplitudes among all events types was conducted.

The statistical analysis and the corresponding data processing are further detailed in e-Figure S1 in Supplementary Material. Significance level was set at highly stringent criteria (*p* = 0.001) for null-hypothesis testing.

## Results

### Characteristics of the Study Population, Scoring, and Signal Processing Results

30 subjects were enrolled of whom 25 subjects had at least 4 h of sleep along with good quality signals. Patient characteristics are summarized in Table 1 and were representative of the broad spectrum of OSA severity. The final database comprised a total of 154 h of recordings with 2,092,157 paired observations of MM and EMG-d amplitudes.

**Table 1:** Clinical and polysomnographic characteristics of 25 patients.

Parameter	Mean	SE	95% CI
Age (years)	49.7	2.5	30.94 to 69.67
BMI (kg/m^2^)	30.0	1.3	20.86 to 39.57
Epworth score	11.1	0.9	5.00 to 18.80
TST (min)	370.0	18.3	276.70 to 486.40
REM.ST (%)	13.5	2.0	0 to 30.1
MAI (n/h)	24.8	2.6	10.58 to 48.04
AHI (n/h)	24.8	5.2	4.18 to 76.92
RERAI (n/h)	4.6	0.7	0.02 to 11.58
ODI (n/h)	16.0	5.2	0.00 to 71.48

*TST, total sleep time; REM.ST, total REM sleep time; MAI, micro-arousal index; AHI, apnea hypopnea index; RERAI, Respiratory Effort Related Arousal index; ODI, oxygen desaturation index*.

### Cross-Correlations between the Amplitudes of EMG-d and MM Signals

Overall, MM and EMG-d amplitudes were significantly cross-correlated in the 28,211 randomized PSG segments with a median correlation coefficient of 0.63 (95% CI: 0.20–0.94) and a median lag of 0.0 s (95% CI: −1 to +1 s). Cross-correlations coefficients calculated on each fragment of normal or disordered breathing are reported in the Online Supplement (e-Table S1 in Supplementary Material).

### Relationship between EMG-d Activity and MM Interaction of REM Sleep on This Relationship

**Figure 3:**
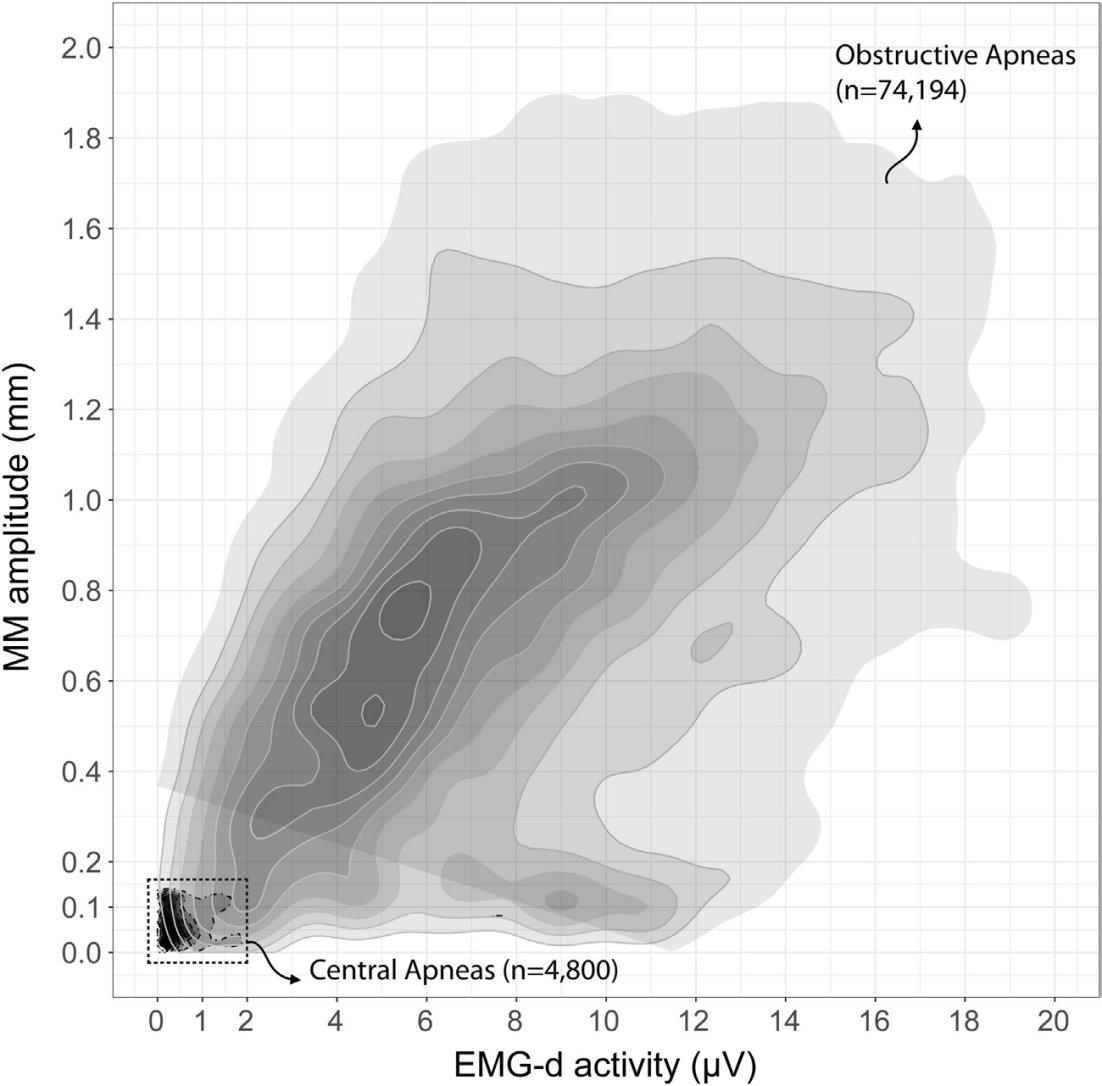
Two-dimensional density plot of Mandibular movements (MM) and diaphragmatic electromyography (EMG-d) amplitudes during central and obstructive apneas. Two-dimensional density area was separately determined for central apnea (*n* = 4,800, discontinued borderline, black filled area at lower left corner) and obstructive apnea (*n* = 74,194, continuous gray borderline). This figure represents the combined information on the association pattern between MM and EMG-d signals and the Gaussian Kernel density of these two variables.

Mandibular movements amplitude was significantly associated with EMG-d activity during normal and disordered breathing (obstructive apneas, hypopneas, and RERA). A 2D-density plot (Figure 3) shows a positive linear relationship between MM and EMG-d amplitudes during obstructive apneas (*n* = 74,194) compared to central apneas (*n* = 4,800).

Based on the mixed linear model (Table 2), mean MM amplitude increased by 0.28 mm for each increase in EMG-d activity of 10 µV (*p* = 0.0004). The REM sleep state reduced the mean MM amplitudes by 0.17 mm. However, the relationship between EMG-d and MM amplitudes remained positive during REM sleep stages. No significant interactions between REM sleep and EGM-d emerged (*p* = 0.056).

**Table 2:** Fixed effects in mixed linear model.

	Fixed effects		
	Estimated (mm)	95% CI (mm)	*p*
Intercept	0.259	0.143 to 0.376	0.00001
EMG-d activity	0.028	0.012 to 0.043	0.0005
REM	−0.174	−0.302 to −0.045	0.007
Interaction effect (EMG-d: REM)	0.004	0.009 to 0.017	0.056

The table represents the fixed effects of EMG-d activity, REM sleep stage (as binary factor), and interaction between them in a random slope model. Model’s formula: MM ~ Intercept + 0.028*(diaphragm EMG) − 0.174*(if sleep stage is REM) + 0.004*(diaphragm EMG during REM) + Random Error ~ ((diaphragm EMG during REM) | subject identity). Model’s goodness of fit (*F*-test): *p*-value < 0.00001; median of residual errors: −0.097 (CI: −0.353 to 0.211). Units of MM and EMG-d amplitudes are millimeter and microvolt, respectively.

### Association between EMG-d Activity and MM Amplitude during Normal Sleep or Disordered Breathing Events

**Figure 4:**
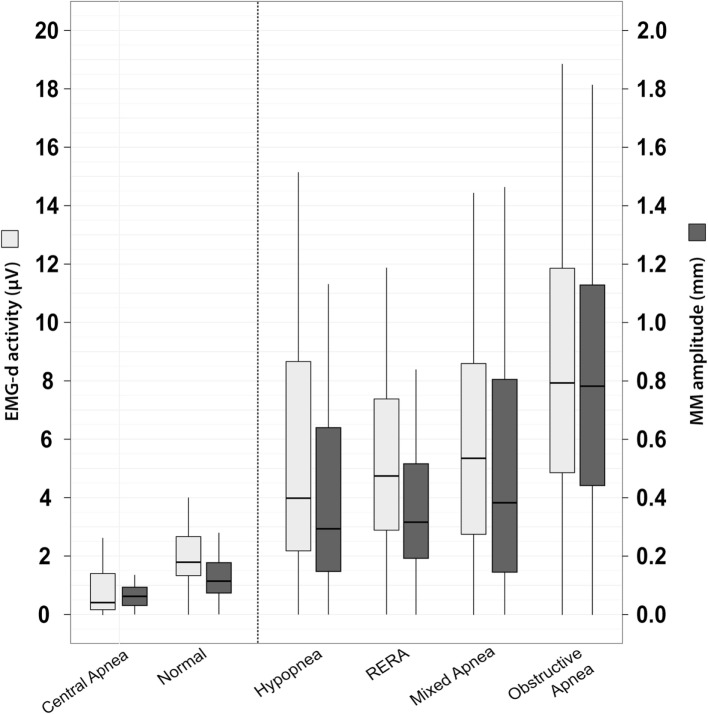
Diaphragm EMG (EMG-d) activity and mandibular movements (MM) amplitudes among normal sleep and sleep breathing disorders. Tukey boxplots represent the median, interquartile ranges, minimum, and maximum values of EMG-d activity (gray) and MM amplitude (black) during normal and five types of sleep disordered breathing. Events ranking was based on median values of MM and EMG-d amplitudes. Numerical values of these medians are presented in e-Table S2 in Supplementary Material (Online Supplement). The pairwise quantile comparison using Harrell–Davis method indicates significant differences in median, lower, and upper quartiles of both MM and EMG-d amplitudes across the six groups.

Figure 4 shows the medians of EMG-d and MM amplitudes according to the various different respiratory events. MM amplitudes increased proportionately to EMG-d from normal breathing to obstructive apnea events. The highest amplitudes of MM and EMG-d were observed during obstructive apneas and the lowest during central apnea episodes. This stratification of EMG-d activity among the different events closely matched with that observed with MM amplitudes.

## Discussion

This is the first study showing good agreement between diaphragmatic activity objectively measured *via* endoesophageal EMG and MM when evaluating RE in patients in the context of SDB. For each 10 μV increase in EMG-d activity, MM amplitude increased by 0.28 mm MM. This association remained valid even during REM sleep, during which EMG-d and MM amplitudes were obviously reduced.

### MM: An Innovative, Non-Invasive Accurate Signal for Measuring REs during Sleep Studies

The EMG signal corresponding to diaphragmatic activation is a well established reference method for quantifying different levels of RE during sleep-associated breathing events. As would be anticipated, EMG-d activity was highest during obstructive apnea events (Figure 4). The measured values of EMG-d represent the intensity of motor unit recruitment and/or firing rate at the level of the crural diaphragmatic sarcolemma and reflect the intensity of the neural respiratory drive to the diaphragmatic musculature (15, 19). EMG-d was, therefore, markedly reduced during central apneic events in comparison with epochs free of any respiratory disturbance. EMG-d was in the intermediate range during RERAs, hypopneas, and mixed apnea events. MM amplitudes accurately identified RE during SDB, and in this respect, MM performance was similar to EMG-d signals (Figure 4). The REM-related reduction in amplitude of MM did not suppress the high degree of concurrence with MM-EMG-d, a feature that represents a major advantage compared to other non-invasive techniques, such as pulse transit time or inspiratory flow limitation assessed by nasal pressure recordings (27, 28). REM sleep is associated with marked physiological variations in RE and airflow, which makes identification of the classical pattern of flow limitation more difficult. REM sleep is also associated with marked autonomic changes, leading to baseline PTT values to markedly fluctuate, thereby making interpretation difficult (9, 26). A non-invasive tool such as measurement of MM becomes, therefore, particularly interesting in two clinical situations during which it is difficult to separate upper airway obstructions from decreases in ventilatory drive: (i) neuromuscular disorders with pseudo central events owing to diaphragm weakness, and REM-related atonia (29); and (ii) residual events under non-invasive ventilation (30).

### Physiological Background and MM: Novel Potential Insights from the Current Study

The physiological relationship between RE and MM can be viewed as follows: the forces generated by the displacement of the thorax are transmitted through the mediastinum to the upper airways. During each breath, the negative swing of the intrathoracic pressure exerts a caudal traction stretching and favoring the patency of the upper airway (31). The position of the mandible will change at the breathing frequency of the tracheal movements as they apply traction on the hyoid arch and, therefore, generate a corresponding traction on the submental muscles (the so called “tracheal tug”) (32). However, REM sleep stage has a fixed effect of MM amplitude reduction reflecting the reduction in neural respiratory drive to the respiratory musculature associated with this sleep stage. Beyond central respiratory drive originating in the brainstem impacting motoneurons innervating the diaphragm, there are active nuclei involved in the recruitment and relaxation of muscles controlling MM (33). These nuclei are also independently subjected to REM sleep atonia (34). Our current findings suggest that MM can both reflect RE in the context of pharyngeal obstruction, but also during variations in respiratory drive, e.g., during central apnea or during the crescendo ventilation period of periodic breathing (see e-Figure S2 in Supplementary Material). In this situation, the mandible moves at the breathing frequency highly synchronous with the thorax (35).

### Limitations of the Study

In this study, we did not differentiate between central and obstructive hypopnea events. PSGs were scored blindly regarding MM and EMG-d and such distinction between central and obstructive hypopnea was for some events difficult to perform in the absence of a quantitative assessment of RE; therefore, we elected to simply score all hypopnea events, which is recommended as optional in the AASM rules manual (1).

Other movements of the mandible (due to bruxism, chewing, or swallowing) have been described during sleep, but do not occur at the breathing frequency and are thus easy to identify (36).

A long distance (>15 cm) between the position of the magnetometers could reduce the signal to noise ratio and degrade the overall performance of magnetometry. However, in the cohort reported here, as well as in our extensive experience with MM assessments, the maximal mouth opening was such that this critical distance was never reached during any of the recordings; of note, attention to the parallelism or the midsagittal placement of the magnetometers also is also important to avoid degradation of the MM signals (20).

## Conclusion

The changes in amplitude of mandibular respiratory movements during sleep closely parallel the variations in diaphragmatic EMG activity and allow for accurate identification of RE and respiratory drive. Monitoring of MM signals provides useful information about the obstructive nature of sleep disordered breathing and REM sleep changes in ventilatory drive.

## Glossary

MM: mandibular movements (unit: millimeter).

EMG-d: Diaphragmatic electromyography (RMS signal unit: microvolt).

SDB: sleep time period with disordered breathing consisting in obstructive apnea, mixed apnea, central apnea, hypopnea, or Respiratory Effort Related Arousal (RERA).

## Ethics Statement

The study was approved by the local human ethics committee (IRB 00004890—number B707201523388). All participants provided a written informed consent.

## Author Contributions

J-CB and J-BM designed research; J-BM, VC, and SD performed research; J-BM, N-NL-D, J-CB, HG, PS, and J-LP analyzed the data. J-BM, N-NL-D, J-CB, PS, DG, and J-LP wrote the paper. J-CB, DG, and J-LP revised and edited the final manuscript.

## Conflict of Interest Statement

The authors declare that the research was conducted in the absence of any commercial or financial relationships that could be construed as a potential conflict of interest.
