# Supplementary material for: Mandibular Movements As Accurate Reporters of Respiratory Effort during Sleep: Validation against Diaphragmatic Electromyography
Source: Front Neurol. 2017 Jul 21;8:353. doi: 10.3389/fneur.2017.00353 (PMC5519525; doi:10.3389/fneur.2017.00353)
Supplement: Supplementary file 1 [file data_sheet_1.docx]

**Online Supplement.**

**e-Figure 1**: Data processing for Statistical Analysis.


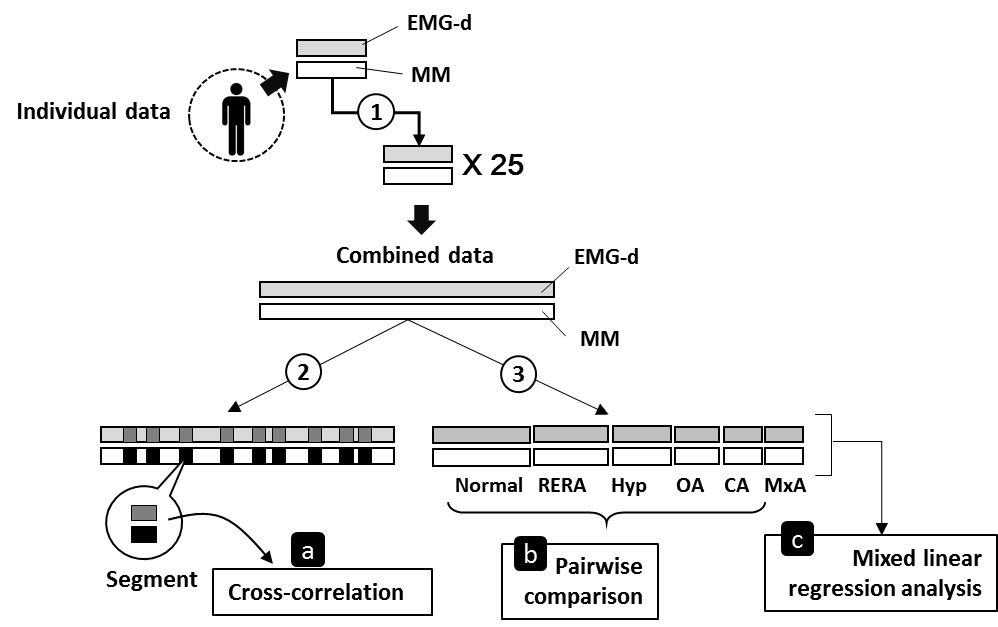


Note-sequence of analysis: (1) Individual paired data prepared for analysis, including exclusion of periods of arousal/microarousal, size reduction at 10Hz, noise-reduction, envelope treatment and compilation. The combined dataset consists of 2,092,157 paired values (10.1 % REM, 89.9 % NonREM) of MM and EMG-d amplitudes; (2) Bootstrapping of 28,211 segments made of continuous values. Each segment during the period of interest was data equally sized depending on the median duration of the related event. Each period of event was represented by about 4600 segments; (2a). A Cross Correlation Function on each segment was performed. Coefficients were averaged. (3b) Medians of MM and EMG-d amplitudes were compared among the 6 entire subsets of Normal breathing (Normal, n=497,828 or 23.8%), Central apnea (CA, n=4800 or 0.2%), Obstructive hypopnea (Hyp, n=539,194 or 25.8%), Obstructive apnea (OA, n=74,194 or 0.35%), RERA (n=945,684 or 45.2%), Mixed apnea (MxA, n=30,457 or 0.15%) using Harell-Davis quantile estimator based method. A visual data exploration by 2D-density plot based on MM and EMG-d amplitudes from Central apneas (n= 4800) and Obstructive apneas (n=74,194) is shown in Figure 3. (3c) Mixed linear regressions were performed on the whole dataset.

**e-Table 1:** Cross-correlation analysis on random sampled fragments

| Events | Number of fragments | Cross-correlation coefficient | |
| --- | --- | --- | --- |
|  |  | Median | 95%CI |
| Normal breathing | 4975 | 0.60 | 0.34 to 0.85 |
| Central apneas | 4677 | 0.34 | 0.10 to 0.79 |
| RERA | 4720 | 0.41 | 0.18 to 0.76 |
| Hypopneas | 4851 | 0.73 | 0.43 to 0.95 |
| Obs. Apneas | 4446 | 0.80 | 0.49 to 0.78 |
| Mixed apnea | 4542 | 0.76 | 0.47 to 0.94 |

Caption: The table represents the median and 95%CI of cross-correlation coefficient estimated in 4542 to 4975 randomised, equal sized and continuous fragments for each type of sleep breathing disorders. Pairwise comparison by Harrell-Davis method showed a significative difference in cross-correlation coefficients among the 6 groups.

**e-Table 2**: Bootstrapped medians of MM and EMG-d amplitudes during normal and 5 types of sleep disordered breathing.

| Events | MM (mm) | EMG-d (µVolt) |
| --- | --- | --- |
|  | Bootstrapped Median (95%CI) | Bootstrapped Median (95%CI) |
| Normal breathing | 0.114 (0.113 to 0.114) | 2.105 (2.102 to 2.108) |
| Central apnea | 0.062 (0.060 to 0.063) | 0.412 (0.393 to 0.428) |
| RERA | 0.319 (0.318 to 0.320) | 4.824 (4.817 to 4.834) |
| Hypopnea | 0.303 (0.302 to 0.304) | 4.174 (4.161 to 4.189) |
| Obstructive Apnea | 0.844 (0.840 to 0.847) | 8.227 (8.183 to 8.271) |
| Mixed apnea | 0.410 (0.404 to 0.414) | 5.444 (5.395 to 5.500) |

**e-Figure 2 : Polysomnographic 3 min segment including MM and EMG-d signals during episodes of central apneas (A) and periodic breathing with a crescendo – decrescendo pattern (B)**

**A**

**
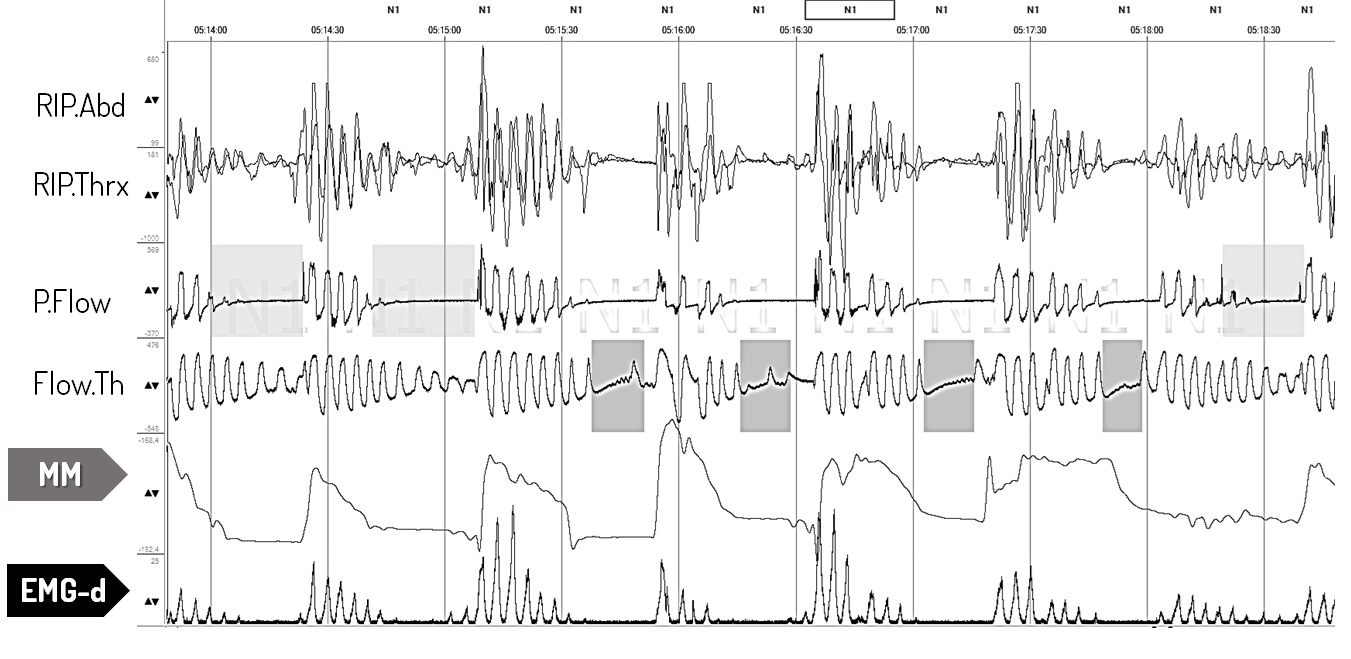
**

**B**

**
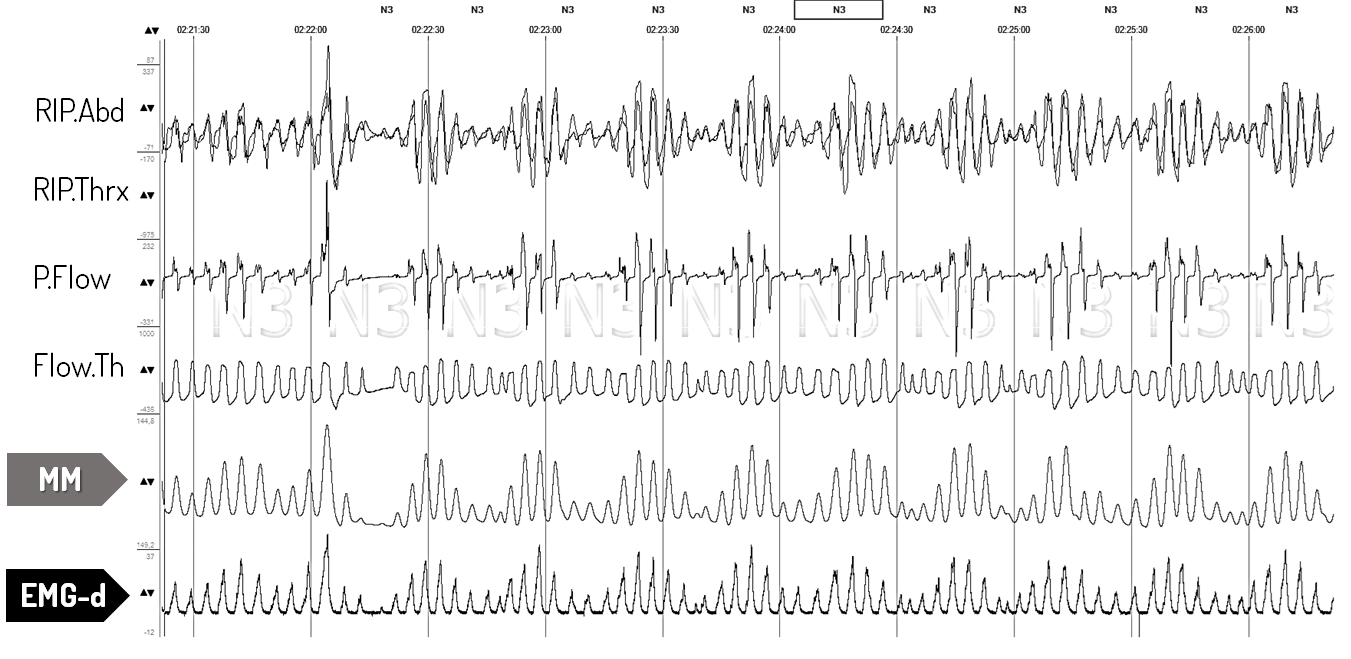
**

Caption: RIP. Abd, RIP. Thx: abdominal and thoracic inductance belts; P. Flow, Flow. Th: nasal pressure transducer and oronasal thermal flow sensor; MM: mandibular movements; EMG-d: diaphragmatic EMG activity.
